# Insulin-like growth factors are essential to prevent anoikis in oestrogen-responsive breast cancer cells: importance of the type I IGF receptor and PI3-kinase/Akt pathway

**DOI:** 10.1186/s12943-015-0482-2

**Published:** 2016-01-22

**Authors:** Brendan C. Luey, Felicity E. B. May

**Affiliations:** Northern Institute for Cancer Research and Newcastle University Institute for Ageing, Department of Pathology, Faculty of Medical Sciences, University of Newcastle upon Tyne, Framlington Place, Newcastle upon Tyne, NE2 4HH UK

**Keywords:** Akt PKB, mitogen-activated protein kinase (MAPK), anoikis, insulin-like growth factor (IGF), type I IGF receptor, oestrogen-dependent breast cancer, apoptosis, integrin, metastases, focal adhesion kinase (FAK)

## Abstract

**Background:**

Detachment of epithelial cells from the extracellular matrix initiates programmed cell death by a process termed anoikis. Malignant cells must acquire anoikis resistance to leave the primary tumour and metastasise. Multiple signal transduction pathways can activate anoikis and confer anoikis resistance, but these are not understood in breast cancer.

**Methods:**

Models for anoikis of oestrogen-responsive breast cancer cells were established and the protective effects of IGF-1 tested. Cleaved PARP was measured by western transfer and cleaved caspase 3 by flow cytometry. Pathways involved in anoikis and in anoikis resistance were investigated with PI3-kinase, Akt, and MEK1 and MEK2 inhibitors. The importance of the type I IGF receptor was investigated by IGF-concentration dependence, siRNA knockdown and pharmacological inhibition. Association between *IGF-1R* expression and relapse with distant metastasis was analysed in 1609 patients by log rank test.

**Results:**

Unattached breast cancer cells required culture in serum-free medium to induce anoikis. Rapid loss of FAK, Akt and Bad phosphorylation was concurrent with anoiks induction, but ERK1 and ERK2 phosphorylation increased which suggested that anoikis resistance is mediated by the PI3-kinase/Akt rather than the Grb2/Ras/MAP-kinase pathway. IGF-1 conferred anoikis resistance in serum-free medium. IGF-1 activated the PI3-kinase/Akt and Grb2/Ras/MAP-kinase pathways but experiments with PI3-kinase, Akt and MEK1 and MEK2 inhibitors showed that IGF protection is *via* the PI3-kinase/Akt pathway. The concentration dependence of IGF protection, knockdown experiments with siRNA and pharmacological inhibition with figitumumab, showed that IGF-1 signals through the type I IGF receptor. The crucial role of the type I IGF receptor was demonstrated by induction of anoikis in full serum by figitumumab. High *IGF-1R* expression was associated with reduced time to relapse with distant metastases in oestrogen receptor-positive patients, especially those with aggressive disease which confirms its relevance *in vivo*.

**Conclusions:**

Anoikis resistance of oestrogen-responsive breast cancer cells depends upon IGF activation of the type I IGF receptor and PI3-kinase/Akt pathway. Because IGF-dependent evasion of anoikis will facilitate metastasis by malignant breast cancer cells, effective inhibition of IGF signal transduction should be included in combinations of targeted drugs designed to treat metastatic oestrogen receptor-positive breast cancers.

## Background

Worldwide, more than 1.7 million women are diagnosed annually with breast cancer of whom at least 500,000 die as a result of metastatic disease [1]. Metastasis necessitates release of malignant cells from the primary tumour and their movement to and establishment at distant sites. Normal breast epithelial and myoepithelial cells attach to each other and to the extracellular matrix. Loss of these attachments induces programmed cell death in a process called anoikis [2–4]. Breast cancer cells must become resistant to anoikis as they invade breast and surrounding tissue, intravasate into blood and lymphatic vessels and metastasise [5]. Blockade of the pathways responsible for anoikis resistance offers a powerful strategy for the elimination of metastatic cells.

Integrins are transmembrane proteins that provide connections between extracellular matrix proteins and the actin-based cytoskeleton in normal tissues. Integrins transduce signals via sub-membrane, focal adhesion protein complexes, called focal adhesions, which connect integrins with signal transduction proteins [6, 7]. Integrin engagement activates focal adhesion kinase (FAK) to suppress anoikis [3] by transmission of cell survival signals through multiple signal transduction pathways [3, 8].

The effects of insulin-like growth factors (IGFs) are mediated by the transmembrane type I IGF or insulin receptors and multiple intracellular signal transduction pathways [9]. Work with transgenic animals implicates IGFs in carcinogenesis [10] and they are significant regulators of breast cancer cell proliferation and invasion [11–15]. Consequently, the IGF signal transduction pathway has been identified as a therapeutic target and inhibitors of the type I IGF receptor have been developed by pharmaceutical companies [9, 16–21].

IGFs confer anoikis resistance in embryonic fibroblasts that have been engineered to overexpress the type I IGF receptor [22] but the importance of IGFs in anoikis resistance and the mechanism by which they might act in oestrogen-responsive breast cancer is unknown. Similarly, the signal transduction pathways involved in the induction of anoikis and in anoikis resistance of breast cancer cells have not been determined.

There have been a few studies that purport to examine the effects of the IGF signal transduction pathway upon anoikis breast cancer cells. However, caspase-dependent programmed cell death was not measured in these studies. In immortalized, normal MCF710A cells that have been modified to overexpress the type I IGF receptor, PTK6 increased signal transduction through the type I IGF receptor and IRS-1 increased the number of viable cells grown in unattached conditions [23]. Another study reported that effects of IGF on the ratio of isoforms of the C/EBPβ protein reduced the proportion of unattached MCF710A cells in sub-G1 phase [24]. Disruption of the type I IGF receptor signal transduction pathway decreased numbers of viable cells of a metastatic variant of MDA-MB-435 breast cancer cell line grown as unattached cells [14]. Another study reported that activation of the p53 pathway after MCF-7 cell detachment leads to a caspase-independent reduction in mitochondrial activity, and that calveolin may reverse the reduction *via* an increase in type I IGF receptor [25]. Thus, despite the impression conveyed in the titles or abstracts of these articles, there have been no studies of the effects of IGFs on anoikis in human breast cancer cells.

We have shown that IGFs inhibit apoptosis in triple-negative breast cancer cells [12] which suggested that they could protect against breast cancer cell anoikis and that blockade of the IGF signal transduction pathway might offer a strategy for promoting anoikis and reducing metastasis. The overall aim of the current study was to investigate the mechanisms by which oestrogen-responsive breast cancer cells evade anoikis. We established an *in vitro* model of anchorage-independent, caspase-dependent cell death and investigated the changes in intracellular signal transduction involved, whether IGF-1 protects the cells from anoikis and the receptor and signal transduction pathway through which IGFs act.

## Results

### Model of anoikis in oestrogen-responsive breast cancer

**Fig. 1:**
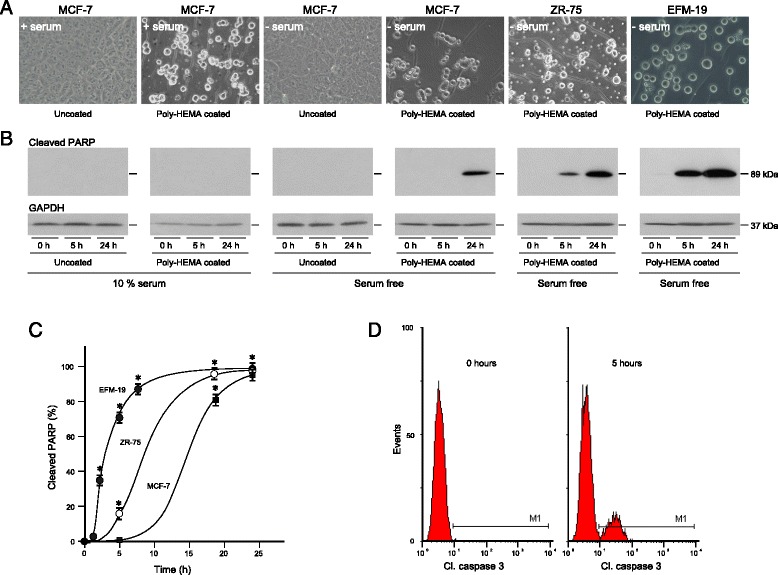
Caspase-dependent programmed cell death of unattached oestrogen-responsive breast cancer cells. MCF-7, ZR-75 and EFM-19 cells were cultured in maintenance medium, trypsinised and added to uncoated or poly-HEMA-coated 35-mm-diameter wells in maintenance medium (10 % serum) or serum-free medium (Serum free) and cultured for 24 h (**a**). Cells were lysed after the indicated times and 10 μg protein aliquots were electrophoresed on 12 % polyacrylamide gels, transferred to nitrocellulose and the amount of 89 kDa cleaved PARP and GAPDH measured by western transfer analysis. Representative western transfer images are shown (**b**). The amount of each protein was determined by densitometric scanning of X-ray films. The amount of cleaved PARP was corrected for GAPDH expression with Labworks 4 software and is expressed as the percentage of the maximum value measured for each cell line (**c**). The mean values ± SEM are shown. Asterisks show times at which there is statistically significantly more cleaved PARP in the unattached cells than in attached cells (ANOVA, p < 0.01). EFM19 cells were incubated in serum-free medium in poly-HEMA-coated 22-mm-diameter wells, fixed, permeabilised and incubated with FITC-conjugated antibody against activated caspase 3 and analysed in a FACSCalibur as described in the Materials and Methods. Representative histograms are shown (**d**). Each experiment included triplicate samples and was replicated thrice

MCF-7 cells were added to uncoated or poly-HEMA-coated culture wells to prevent cell attachment [26]. After 24 h, cells in the poly-HEMA-coated wells grew as rounded cells in suspension (Fig. 1). To investigate if the unattached MCF-7 cells had undergone programmed cell death *via* the caspase-dependent pathway, we measured the cleavage of PARP into the 89 kDa catalytic and 24 kDa DNA binding subunits which cannot repair single-strand DNA breaks. No cleaved PARP was detected in attached or unattached cells cultured in maintenance medium. Attached cells grown in serum-free medium for 24 h maintained their characteristic polygonal morphology and PARP cleavage was not detected. PARP cleavage was induced, however, in unattached cells after 24 h in serum-free medium. Culture of attached cells in serum-free medium for up to three days did not induce significant cell death (data not shown).

Oestrogen-responsive breast cancer cells, ZR-75 and EFM-19, also lost their characteristic epithelial appearance and grew as rounded cells after 24 h culture in poly-HEMA-coated wells in serum-free medium (Fig. 1A-C). A small amount of cleaved PARP was detected in unattached ZR-75 after 5 h and substantially more after 24 h. Cleaved PARP was detected readily in unattached EFM-19 at 1 h and was almost maximal after 6 h. Anoikis was induced also in T-47D cells after 24 h (data not shown). PARP cleavage was not detected in attached cells grown in serum-free medium.

Caspase 3 is important in the execution phase of caspase-dependent programmed cell death. It is activated by cleavage into 17 kDa and 12 kDa subunits; the presence of which indicate that cells are in the execution stages of programmed cell death. Activated caspase 3 was detected by flow cytometry in less than 1 % of the cells prior to and in 15 % of the cells after induction of anoikis (Fig. 1D; student t-test; p < 0.01) which confirmed that caspase-dependent anoikis had been induced.

### Effect of loss of attachment on signal transduction

**Fig. 2:**
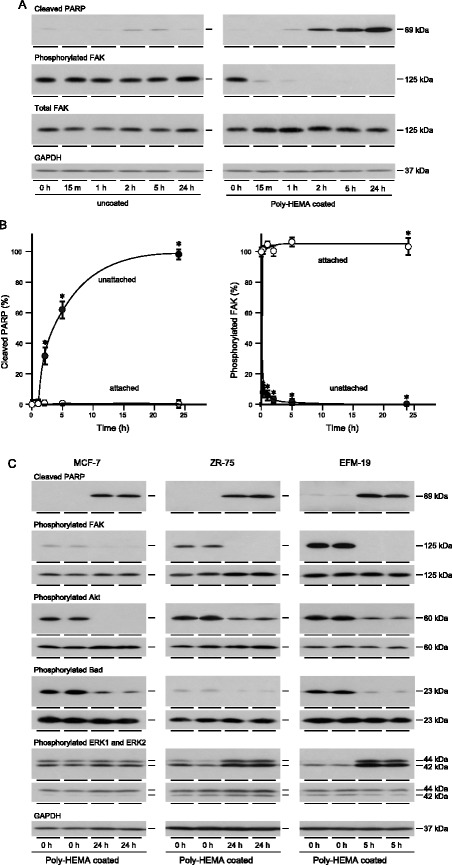
Effect of loss of attachment on activation of signal transduction proteins in breast cancer cells. EFM-19 cells (**a**, **b** and **c**), MCF-7 (**c**) and ZR-75 cells (**c**) were trypsinised, added to 35-mm-diameter cell culture wells that had not (uncoated) or had been coated with poly-HEMA and cultured for the indicated lengths of time in serum-free medium. Cells were lysed and aliquots of 10 μg of protein were electrophoresed on 12 % polyacrylamide gels, transferred to nitrocellulose and the amounts of cleaved PARP, phosphorylated FAK, Akt, Bad, ERK1 and ERK2 and the corresponding total proteins and GAPDH were measured as described in the Materials and Methods. Representative images of the results obtained are shown (**a** and **c**) with the images for each phosphorylated protein above those of the equivalent total protein (**c**). The amounts of cleaved PARP and phosphorylated FAK were determined by densitometric scanning of X-ray films followed by analysis with Labworks 4 software and correction for GAPDH. The results obtained are expressed as a percentage of the maximum amount of cleaved PARP protein measured or phosphorylated FAK measured in attached (o) or unattached (•) cells. The bars show the standard errors of the mean. Asterisks show cleaved PARP that is statistically significantly more, or phosphorylated FAK that is statistically significantly less, in unattached cells than in attached cells (ANOVA, p < 0.01)

To investigate alterations in signal transduction that could account for the anoikic response, EFM-19 cells were grown attached or unattached in serum-free medium for different lengths of time. PARP cleavage was detected after 1 h in the unattached cells and increased thereafter for 24 h (Fig. 2). Little PARP cleavage was detected in attached cells even after 24 h.

The phosphorylation of proteins implicated in anoikis was then measured. There was a dramatic decrease in FAK phosphorylation within 15 min of prevention of cell attachment whereas there was no reduction in FAK phosphorylation in attached cells cultured in serum-free medium for the same length of time. The levels of phosphorylated FAK were minimal by 1 h and decreased further up to 24 h concurrent with the increase in PARP cleavage. There was no concomitant change in the levels of FAK protein. FAK phosphorylation was reduced also in unattached MCF-7 and ZR-75 cultured in poly-HEMA-coated dishes in serum free medium for 24 h. Consistent with a role of the PI3-kinase/Akt pathway in anoikis, Akt phosphorylation was reduced dramatically in MCF-7 and to a lesser extent in ZR-75 and EFM-19. The reduction of Akt phosphorylation led to a reduction in phosphorylation of its downstream target, Bad, in MCF-7 and EFM-19. Reduced phosphorylation of Bad promotes cell death because unphosphorylated Bad sequesters anti-apoptotic Bcl family proteins which prevents their inhibition of pro-apoptotic proteins Bax and Bak.

In contrast to Akt phosphorylation, phosphorylation of ERK1 and ERK2 was increased slightly in MCF-7 and markedly in EFM-19 and ZR-75 cells induced to undergo anoikis which suggests that the Grb2/Ras/MAP-kinase pathway has not been dampened and that it is unlikely to transduce the integrin-dependent cell survival signal. The results are consistent with oestrogen-responsive breast cancer cell attachment activating FAK to signal through the PI3-kinase/Akt/Bad pathway to provide an important cell survival signal in attached oestrogen-responsive breast cancer cells.

### IGFs protect oestrogen-responsive breast cancer cells from anoikis

**Fig. 3:**
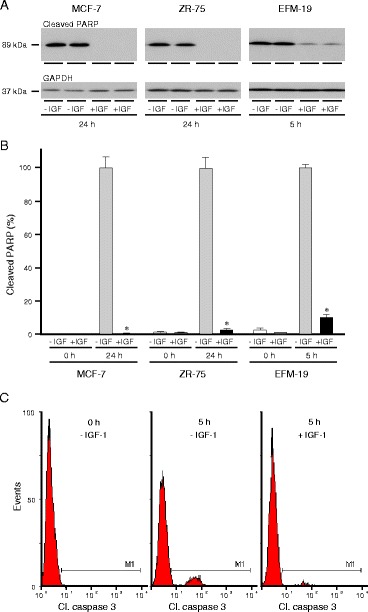
Protective effect of IGF-1 against anchorage-dependent programmed cell death in breast cancer cells. MCF-7 (**a** and **b**), ZR-75 cells (**a** and **b**), and EFM-19 cells (**a**, **b** and **c**) were trypsinised, resuspended in serum-free medium alone or with 50 ng/ml IGF-1, placed in 35-mm-diameter poly-HEMA-coated wells and cultured for the indicated lengths of time. Cells were lysed and aliquots of protein analysed by western transfer for cleaved PARP and GAPDH. Representative western transfer images are shown (**a**). The amount of each protein was determined by densitometric scanning of X-ray films, corrected for GAPDH and is expressed as the percentage of the maximum value for each cell line (**b**). Asterisks show times at which cleaved PARP is statistically significantly less, in the presence of IGF-1 than in its absence (ANOVA, p < 0.001). EFM-19 cells were collected, fixed, permeabilised and incubated with FITC-conjugated antibody against activated caspase 3 and analysed in a FACSCalibur. Representative histograms are shown (**c**). The data was analysed with WinMDI which confirmed the protective effect of IGF-1 (p = 0.003)

Many factors in serum might account for the anoikis resistance of oestrogen-responsive breast cancer cells. To test if IGFs in serum might contribute to the anoikis resistance of unattached oestrogen-responsive breast cancer cells cultured in maintenance medium, cells were cultured in poly(HEMA)-coated wells in serum-free medium in the absence or presence of IGF-1 (Fig. 3). The amount of cleaved PARP detected was lower in cells cultured in the presence of IGF-1 than in its absence (p < 0.001). IGF-1 prevented completely the induction of anoikis in MCF-7, inhibited it thirty-fold in ZR-75 and ten-fold in EFM-19 cells.

The protective effect of IGF-1 on the induction of anoikis was analysed also by measuring activated caspase 3. The proportion of EFM-19 cells with activated caspase 3 in the execution phase of cell death was reduced by 78 % in cells cultured in poly-HEMA-coated dishes in the presence of IGF-1 compared to in cells cultured in its absence (Fig. 3C; p < 0.01), which confirms that IGF-1 protects cells from caspase-dependent anoikis and suggests that they might be important contributors to the resistance conveyed by culture in serum.

**Fig. 4:**
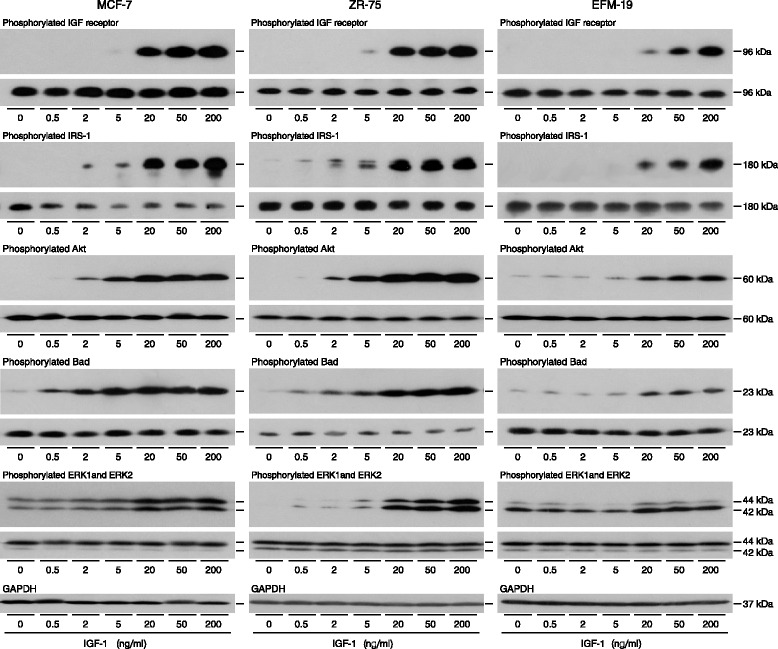
Activation of signal transduction proteins by IGF-1 in oestrogen-responsive breast cancer cells. MCF-7, ZR-75 and EFM-19 cells were cultured for two days in phenol red-free medium supplemented with charcoal-treated serum, for 2 h in serum-free medium and incubated in the absence or presence of the indicated concentrations of IGF-1 for 15 min in the same medium. Aliquots of 10 μg of protein were electrophoresed on polyacrylamide gels, transferred to nitrocellulose and incubated with antibodies specific for phosphorylated IGF receptor, phosphorylated IRS-1, phosphorylated Akt, phosphorylated Bad, and phosphorylated ERK1 and ERK2, antibodies against the equivalent total proteins and developed as described in the Materials and Methods. Representative images from the phosphorylated protein are shown above those of the equivalent total protein

The downstream signal transduction pathways that might be responsible for the protective effect of IGF-1 in oestrogen-responsive breast cancer cells were investigated. IGF-1 stimulated auto-phosphorylation of IGF receptors in all three cell lines (Fig. 4). Activated IGF receptors interact with and induce phosphorylation of adaptor proteins of which IRS-1 is considered to be the most important for signalling *via* both PI3-kinase/Akt and Grb2/Ras/MAP-kinase pathways [27]. IRS-1 phosphorylation was induced by nanomolar concentrations of IGF-1. Phosphorylation of Akt was stimulated by 2 ng/ml IGF-1 in MCF-7 and ZR-75 and by 20 ng/ml IGF-1 in EFM-19, and of Bad by 0.5 ng/ml IGF-1 in MCF-7 and ZR-75 and by 20 ng/ml IGF-1 in EFM-19. Activation by phosphorylation of ERK1 and ERK2 was stimulated most markedly in ZR-75 cells but required higher IGF-1 concentrations, 20 ng/ml, than for the stimulation of Akt phosphorylation. The total levels of the signal transduction proteins did not change during the IGF-1 treatment.

**Fig. 5:**
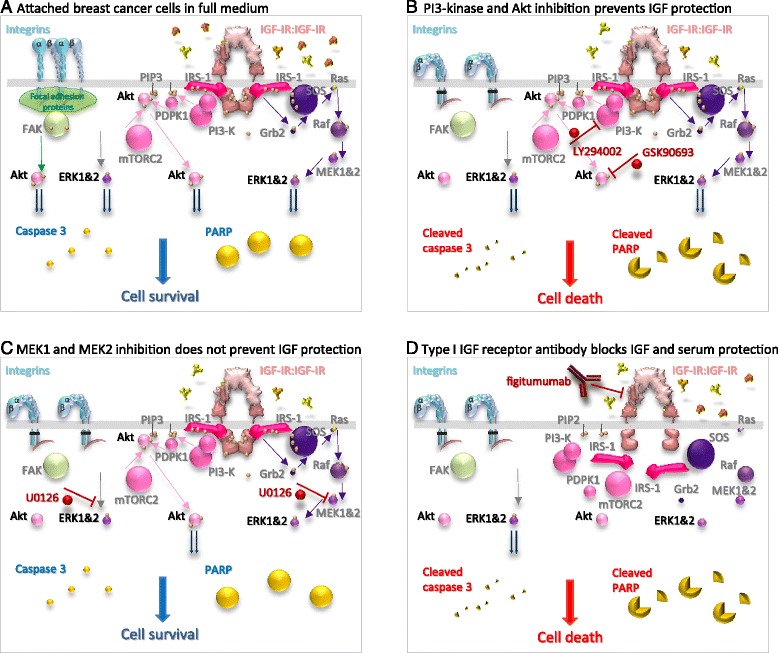
Importance of the type I IGF receptor and PI3-kinase\Akt pathway in the IGF-protection from anoikis. Representations of the alpha and beta integrins (cyan and light purple) and their activation of Akt *via* the focal adhesion and FAK signal transduction pathway (green) (**a**). The homo-tetrameric type I IGF receptor (IGF-IR, pink) has the α-chains of each receptor coloured lighter than the β-chains. The extracellular ligands: insulin (orange), IGF-2 (ochre) and IGF-1 (pale yellow) are shown. Signal transduction from the activated receptor is depicted through IRS-1 *via* the PI3-kinase, PIP3, mTOR2, PDPK1 and Akt pathway (pink) or the Grb2, SOS, Ras, Raf, MEK1 and MEK2, ERK1 and ERK2 pathway (purple). The downward pointing grey arrow indicates constitutive activation of ERK1 and ERK2. The downward pointing metallic blue arrows indicate that a signal has been transduced from each pathway. The pale gold spheres represent phosphorylated moieties. The GTP on activated Ras is a light yellow sphere and the GDP on inactive Ras an ivory sphere. Uncleaved inactive caspase 3 is shown as a small bright yellow sphere and uncleaved active PARP as a large bright yellow sphere. Cleaved active caspase 3 and cleaved inactive PARP are shown as the equivalent fragmented spheres. Downward pointing larger blue arrows indicate that cell survival and downward pointing red arrows indicate that cell death ensues. Kinase inhibitors (brick red) that inhibit PI3-kinase, LY294002, or Akt, GSK 690693, abrogate the protective effect of IGF on cell survival of unattached cells (**b**) whereas the MEK1 and MEK2 kinase inhibitor, U0126, does not (**c**). Inhibition of the type I IGF receptor with the specific inhibitory antibody, figitumumab (brick red), induces anoikis in IGF-stimulated cells or in cells grown in serum (**d**)

These experiments show that oestrogen-responsive cells are protected from cell death by anchorage dependent signals from integrins through FAK and Akt (Fig. 5A). Resistance to anoikis can be mediated through the IGF signal transduction pathway. IGFs activate the PI3-kinase/Akt, and the Grb2/Ras/MAP-kinase signal transduction pathways both of which have been implicated in anoikis resistance.

### Signal transduction pathway that mediates the anti-anoikic effect of IGF-1

**Fig. 6:**
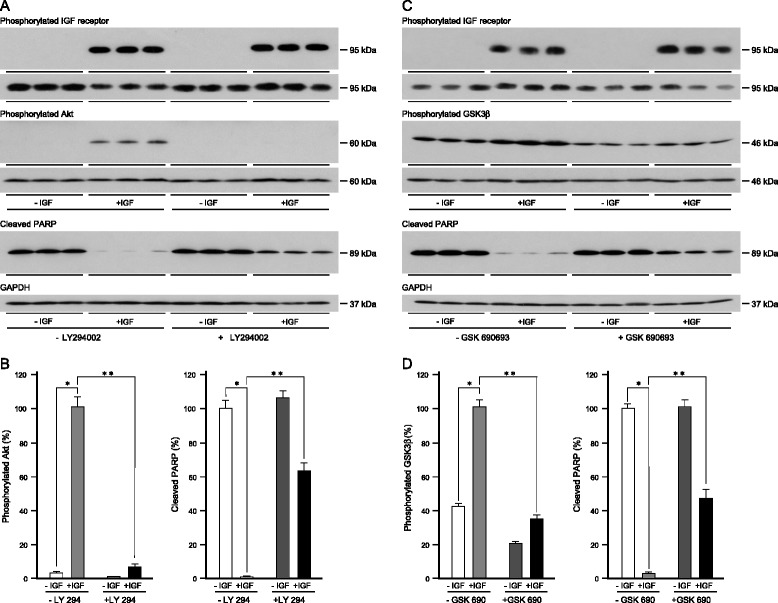
Effect of inhibition of the PI3-kinase/Akt pathway on the protection of breast cancer cells from anoikis by IGF-1. Cells were trypsinised, resuspended in serum-free medium alone or with 50 ng/ml IGF-1 (A and B) or 10 ng/ml IGF-1 (**c** and **d**) and in the absence or presence of 20 μM LY294002 (**a** and **b**) or 100 nM GSK690693 (**c** and **d**) and then placed in 35-mm-diameter cell culture wells that had been coated with poly-HEMA and cultured for 24 h. Cells were lysed and the amount of phosphorylated IGF receptors, phosphorylated Akt, phosphorylated GSK3β, type I IGF receptor, Akt, GSK3β, cleaved PARP and GAPDH were measured by western transfer in aliquots of 10 μg of protein. Representative images are shown with the images for each phosphorylated protein above those of the equivalent total protein (**a** and **c**). The means ± SEM from three experiments are shown. Statistical significance of differences between untreated cells and cells treated with IGF-1 (*), or between cells treated with IGF-1 alone and in the presence of the inhibitor (**) are indicated (p < 0.05; ANOVA)

The importance of the PI3-kinase/Akt pathway in the IGF-dependent anoikis resistance was tested with LY294002, a selective, reversible inhibitor of ATP binding in the catalytic subunit of PI3-kinase [28, 29]. Unattached MCF-7 cells were cultured in serum-free medium in poly-HEMA-coated wells in the absence or presence of IGF-1 and LY294002. Neither the IGF receptors nor Akt were phosphorylated in untreated cells which indicates that there is no signalling through the PI3-kinase/Akt pathway (Fig. 6). Phosphorylation of the IGF receptors stimulated by IGF-1 was not affected by LY294002 whereas IGF-stimulated phosphorylation of Akt was prevented completely. Substantial amounts of cleaved PARP were detected in the untreated cells but very little in the presence of IGF-1. The PI3-kinase inhibitor did not increase anoikis in the absence of IGF-1 which is consistent with this pathway being inactivated completely in unattached cells in serum-free medium. The ability of IGF-1 to prevent cell death was reduced 20-fold by the PI3-kinase inhibitor (ANOVA; p < 0.01).

The role of the PI3-kinase/Akt pathway in IGF-protection of breast cancer cells from anoikis was tested further with GSK690693 [30, 31] a specific, ATP-competitive inhibitor of Akt. Activity of the Akt inhibitor was demonstrated by enhanced Akt phosphorylation in the presence of IGF-1 as has been reported previously (data not shown). Phosphorylation of GSK3β, a downstream target of Akt, was stimulated by IGF-1 (Fig. 6) and GSK690693 inhibited this IGF-stimulated phosphorylation. GSK690693 did not increase anoikis in unattached cells but did inhibit the protective effect of IGF-1 which reinforced the supposition that the IGF-protective-effect is transduced *via* the PI3-kinase/Akt pathway (Fig. 5B).

**Fig. 7:**
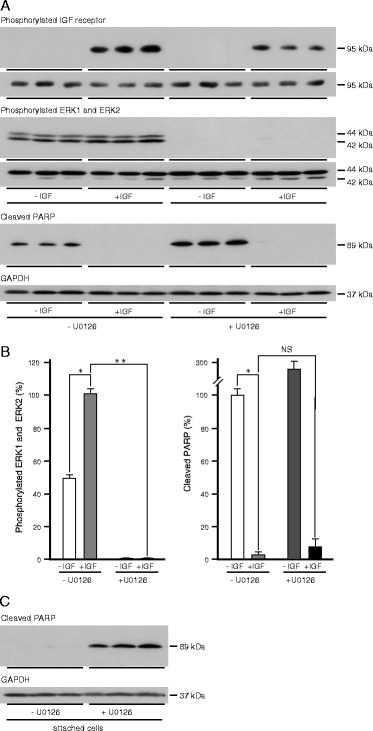
Effect of MAP kinase pathway inhibition on the protection by IGF-1 of breast cancer cells from anoikis. MCF-7 cells were trypsinised, resuspended in serum-free medium alone or serum free medium and 10 ng/ml IGF-1, and in the absence or presence of 1 μM U0126 and then cultured for 24 h in 35-mm-diameter poly-HEMA-coated wells (**a**) or as attached cells (**c**). Cells were lysed and the amount of cleaved PARP, phosphorylated IGF receptors, phosphorylated ERK1 and ERK2, type I IGF receptor, ERK1 and ERK2 and GAPDH were measured by western transfer analysis in aliquots of 10 μg of protein. Representative images are shown with the images for each phosphorylated protein above those of the equivalent total protein (**a**). The means ± SEM from three experiments are shown (**b**). Statistical significance of differences between untreated cells and cells treated with IGF-1 (*), or between cells treated with IGF-1 alone and in the presence of the inhibitor (**) are indicated (p < 0.05; ANOVA)

The contribution of the Grb2/Ras/MAP-kinase pathway to the protection by IGF-1 of oestrogen-responsive breast cancer cells from anoikis was tested with U0126 which is a non-competitive inhibitor of MEK1 and MEK2 [32, 33] that prevents activation of ERK1 and ERK2. Phosphorylated ERK1 and ERK2 were detected in cells in which anoikis had been induced (Fig. 7) which suggests that the Grb2/Ras/MAP-kinase pathway is not inactivated. IGF-1 stimulated receptor phosphorylation, increased ERK1 and ERK2 phosphorylation, and protected cells from anoikis. ERK1 and ERK2 phosphorylation was abrogated completely in the presence of the MEK inhibitor and more cleaved PARP was detected. To investigate if the increased cell death induced by U0126 resulted from increased anoikis or was independent of cell attachment, attached cells were incubated in serum-free medium in the absence and presence of U0126. MCF-7 cultured as attached cells in serum-free medium for up to 24 h did not undergo apoptosis. Apoptosis was induced strongly in the presence of U0126 (Fig. 7C) which indicates that cell death induced by MEK inhibition is independent of cell attachment.

There was no phosphorylated ERK1 or ERK2 in cells incubated in the absence or presence of IGF-1 in the presence of the MEK inhibitor but there was a significant protective effect of IGF-1 against anoikis in the presence of the MEK inhibitor as evidenced by its ability to prevent PARP cleavage. These data demonstrate that the protective effect of IGF-1 against anoikis is not transduced *via* the Grb2/Ras/MAP-kinase pathway (Fig. 5C).

### Importance of the type I IGF receptor

**Fig. 8:**
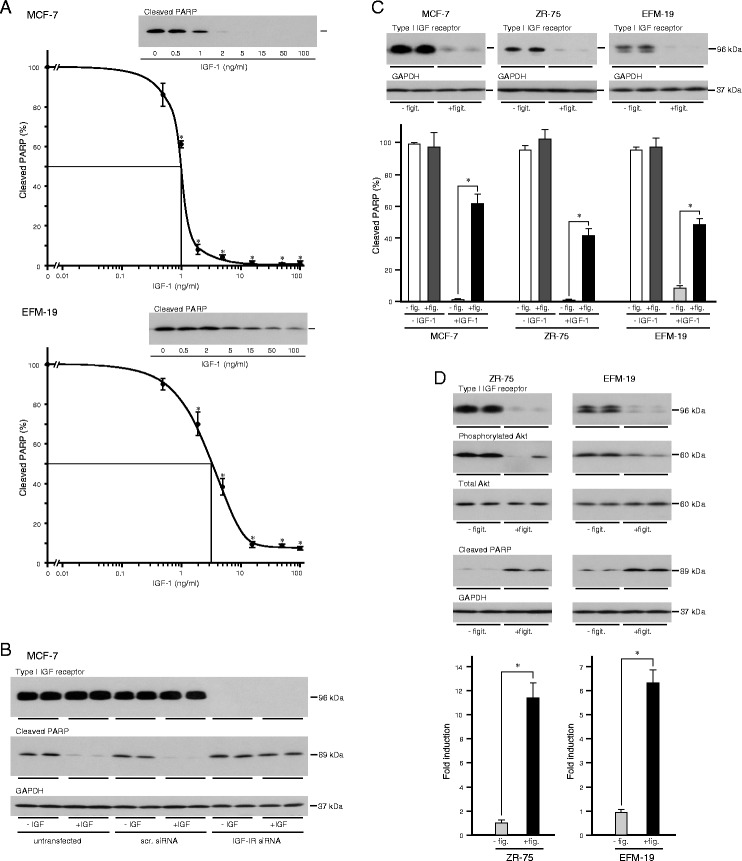
Importance of the type I IGF receptor in IGF protection from anoikis. Cells were trypsinised, resuspended in serum-free medium alone or with the indicated concentrations of IGF-1 (**a**) or in the presence of IgG2 (−figit.) or figitumumab (+figit.) (**c**) or in serum-containing medium in the absence or presence of figitumumab (**d**) and then placed in 35-mm-diameter poly-HEMA-coated wells and cultured for 24 h (MCF-7 and ZR-75) or 5 h (EFM-19). MCF-7 cells were left untransfected or transfected with a scrambled non-silencing siRNA duplex oligonucleotide sequence or an siRNA against type I IGF receptor mRNA and incubated in maintenance medium for 48 h. Cells were trypsinised, resuspended in serum-free medium alone or with 10 ng/ml IGF-1, placed in 35-mm-diameter poly-HEMA-coated wells and cultured for 24 h (**b**). Cells were lysed and aliquots of 10 μg of protein analysed by western transfer for cleaved PARP, type I IGF receptor, phosphorylated Akt, total Akt and GAPDH as described above. Asterisks show PARP cleavage that is statistically significantly less in the presence of IGF-1 than in its absence (**a**) or significantly more in the presence of figitumumab than in its absence (**c** and **d**) (ANOVA, p < 0.01)

Oestrogen-responsive breast cancer cells express relatively more type I IGF receptor than the insulin receptor [12]. IGF-1 has a higher affinity for the former receptor [9, 34]. To investigate which receptor transmits the IGF-protective signal against anoikis, unattached cells were cultured in the presence of different concentrations of IGF-1 (Fig. 8A). There was a small reduction in the amount of cleaved PARP in MCF-7 cells after culture in 0.5 ng/ml IGF-1 compared to in serum-free medium alone, which was significant in 1 ng/ml IGF-1 and was almost complete in the presence of 2 ng/ml IGF-1 and above. IGF-1 was less potent in EFM-19 than in MCF-7 but in both cells lines the protective effect of IGF-1 was maximal at a concentration of 15 ng/ml which indicates that the protective effect of IGF-1 in oestrogen-responsive breast cancer cells is mediated by the type I IGF receptor. The concentrations of IGF-1 at which significant prevention of anoikis was detected coincided with the concentrations that stimulated Akt and Bad phosphorylation (Fig. 4).

Confirmation of the importance of the type I IGF receptor in the IGF-1 protection was sought with siRNA knockdown. Expression of the type I IGF receptor by MCF-7 cells was reduced to undetectable levels after transfection with an siRNA oligonucleotide directed against the type I IGF receptor (Fig. 8B). PARP cleavage in cells cultured in poly-HEMA-coated wells to induce anoikis was inhibited by IGF-1 in untransfected cells and in cells transfected with scrambled oligonucleotide but not in cells in which expression of the type I IGF receptor had been knocked down. The concentration dependence of the IGF-1 anoikis protection, and the inability of IGF-1 to prevent anoikis in cells without type I IGF receptor expression, confirm that the protective effect of IGF-1 against anoikis is transmitted by the type I IGF receptor in these oestrogen-responsive breast cancer cells.

The importance of the type I IGF receptor was corroborated by pharmacological inhibition with the inhibitory antibody, figitumumab. Expression of the type I IGF receptor was barely detectable after incubation with the anti-receptor antibody (Fig. 8C). Incubation of cells with figitumumab reduced significantly the ability of IGF-1 to protect cells from anoikis (Fig. 8C).

To investigate the generality of the importance of the type I IGF receptor and signalling through the IGF transduction pathway in the anoikis resistance of oestrogen-responsive breast cancer cells, unattached cells were incubated in the presence of figitumumab in serum-containing medium. Incubation with figitumumab reduced the amount of the type I IGF receptor and the activation of Akt by phosphorylation of Ser473, which indicates that the type I IGF receptor and the IGF signal transduction pathway are major activators of the PI3-kinase/Akt pathway in oestrogen-responsive breast cancer cells (Fig. 8D).

Importantly, incubation of the cells with figitumumab induced significantly anoikis in the presence of serum. These data demonstrate that abrogation of the IGF-protective effect by pharmacological inhibition of the type I IGF receptor is able to circumvent the protective effects of anti-anoikis factors that are present in serum (Fig. 5B). The effectiveness of figitumumab indicates that the factors that signal through the IGF signal transduction pathway have a major role in the anoikis resistance of oestrogen-responsive breast cancer cells.

**Fig. 9:**
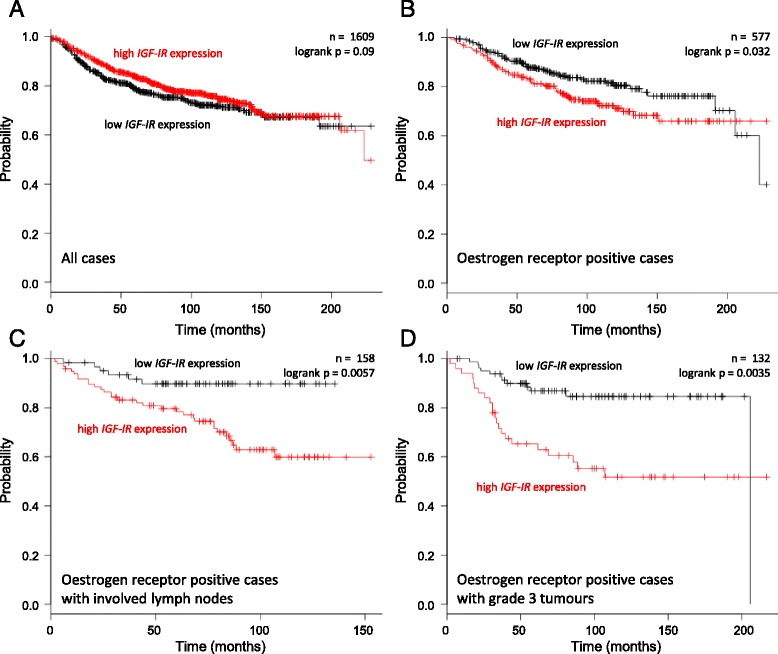
Association of type I IGF receptor expression with distant metastasis-free survival in breast cancer patients. The association between type I IGF receptor gene (*IGF-IR*) expression and time to relapse with distant metastasis was analysed. Kaplan and Meier survival curves are shown for all patients (**a**), patients with oestrogen receptor-positive tumours (**b**), patients with oestrogen receptor-positive tumours and involved lymph nodes (**c**), and patients with oestrogen receptor-positive, grade III tumours (**d**). The differences between the curves was assessed by log rank test and the curves were considered to be significantly different if p < 0.05

The pivotal role of the type I IGF receptor in protection of oestrogen-responsive breast cancer cells from anoikis, which could be critical for the ectopic survival of the malignant cells, suggests that receptor expression might facilitate metastasis. Amongst all patients, there was a trend towards a longer time to relapse with distant metastasis for patients with high type I IGF receptor expression (Fig. 9A) which is consistent with previous studies that have demonstrated survival benefit of high type I IGF receptor expression [35, 36]. However, amongst the sub-group of patients with oestrogen receptor-positive breast cancer, those with higher type I IGF receptor expression had shorter time to relapse with distant metastasis than those with lower expression (log rank; p = 0.032) (Fig. 9B). The difference in time to relapse between patients with high and low receptor expression was even more significant in the subgroup of patients with oestrogen receptor-positive tumours and lymph node involvement (p = 0.0057), and was most significant in patients with oestrogen receptor-positive, grade 3 tumours (p = 0.0035) (Fig. 9C and D).

## Discussion

The majority of breast cancer deaths are caused by the effects of distant metastases in vital organs. Metastatic malignant cells accrue a plethora of characteristics in distinct phases: metastasis initiation, progression and virulence [5]. They must be able to survive without normal cellular and cell matrix attachments to initiate metastasis. The development of resistance to anoikis, the apoptotic process triggered by inappropriate or absence of cell adhesion is an important facet of this transition.

Improvements in the management and survival of breast cancer patients and identification of therapeutic targets will be facilitated by a more profound understanding of metastasis. To this end, we have established a model of oestrogen-responsive breast cancer cell anoikis. Anoikis can be mediated through extrinsic, intrinsic or caspase-independent apoptotic pathways [37, 38]. There have been limited studies on anoikis in breast cancer. Breast cancer cells as exemplified by Hs578T, MDA-MB-231, BT-474 and T-47D have been reported to be inherently resistant to anoikis and it was concluded that this resistance does not involve the PI3-kinase/Akt or Grb2/Ras/MAP-kinase pathways [39]. In contrast, our experiments have identified conditions that allow analysis of breast cancer cell anoikis resistance and have highlighted the importance of the intrinsic apoptotic pathway.

Several signal transduction pathways implicated in anoikis emanate from the focal adhesion complex in response to integrin-mediated cell adhesion to the extracellular matrix. Amongst these, phosphorylated FAK interacts with Grb2 and PI3-kinase [40–42] to activate the Ras/MAP-kinase and Akt pathways, respectively [41, 43]. Our data emphasise the importance of the PI3-kinase/Akt pathway in oestrogen-responsive breast cancer as the rapid and dramatic loss of FAK phosphorylation within 15 minutes of detachment, followed by PARP cleavage after 1 hour, was associated with a decrease in phosphorylated Akt but an increase in phosphorylated MAP-kinases. Consistent with involvement of the intrinsic pathway, Bad phosphorylation decreased concomitantly with phosphorylated Akt and caspase 3 was activated. Dephosphorylated Bad forms heterodimers with Bcl-2 and Bcl-XL, thereby preventing them from interacting with and inhibiting Bax and Bak, which would otherwise form pores in the mitochondrial membrane to release cytochrome c and trigger cell death [38].

Our data show that breast cancer cell culture medium contains factors that confer anoikis resistance and that the major factor is a ligand that signals through the type I IGF receptor. A protective effect of IGF-1 against anoikis was demonstrated first in mouse embryonic fibroblasts engineered to overexpress the type I IGF receptor [22]. As far as we are aware, this is the first demonstration that IGFs protect breast cancer cells from caspase-dependent anoikis.

Many mechanisms are suggested to explain how malignant cells that detach from primary tumours evade cell death. These include changes to integrin expression [44], hypoxia which induces ligand-independent activation of growth factor receptors and redox-mediated decrease of pro-apoptotic factors [45], and EMT activation [46]. Our results demonstrate that oestrogen-responsive breast cancer cells avoid anoikis *via* a prosurvival pathway in which the type I IGF receptor is activated by IGFs. The type I IGF receptor is expressed widely on breast cancer cells and mediates the effects of IGFs on cell migration [13, 47] and proliferation [11, 12, 48]. IGF-1 activated both the PI3-kinase/Akt and Grb2/Ras/MAP-kinase pathways but our experiments with PI3-kinase, Akt and MEK inhibitors establish that anoikis resistance is conferred preferentially through the PI3-kinase/Akt pathway.

Although the Grb2/Ras/MAP-kinase pathway is not involved in the anoikic resistance of oestrogen-responsive breast cancer cells, its inhibition induces significant cell death which is abrogated almost completely by IGF-1. IGF-1 overcomes the cell death signal induced by inhibition of MEK1 and MEK2 without an increase in MAP-kinase phosphorylation. These results suggest that inhibition of the Grb2/Ras/MAP-kinase pathway is unlikely to be effective in the treatment of oestrogen-responsive breast cancer unless treatment is combined with inhibition of IGF signal transduction.

IGFs may signal through the type I IGF receptor, through isoform A and, to a lesser extent, through isoform B of the insulin receptor [9, 34, 49]. The receptor involved is of clinical importance because drugs such as figitumumab, cixutumumab, ganitumab and dalotuzumab are directed specifically against the type I IGF receptor [16–19] whereas BMS-754807 and linisitinib inhibit both the type I IGF and insulin receptors [20, 21]. Our data indicate that signalling through the type I IGF receptor is dominant in oestrogen-responsive breast cancer.

## Conclusions

We have established a reliable model of caspase-dependent anoikis for oestrogen-responsive breast cancer cells. We demonstrate the importance of the intrinsic pathway in anoikis and that IGF-1 can reinstate anoikis resistance of unattached oestrogen-responsive breast cancer cells cultured in serum-free medium. IGF-1 activated both the PI3-kinase/Akt and Grb2/Ras/MAP-kinase pathways but our experiments with PI3-kinase, Akt and MEK inhibitors show that anoikis resistance is conferred through the PI3-kinase/Akt pathway. Although ERK1 and ERK2 are not important in IGF-dependent anoikis-resistance, IGF-1 is able to circumvent apoptosis induced by inhibition of MEK1 and MEK2 without any increase in MAP-kinase phosphorylation. The IGF-1 signal is transduced *via* the type I IGF receptor and incubation with type I IGF receptor specific antibody, figitumumab, induces anoikis of cells grown in serum. This is the first demonstration of the importance of IGFs and the type I IGF receptor in the resistance of oestrogen-responsive breast cancer cells to caspase-dependent anoikis. The importance of the type I IGF receptor is supported by the association of higher receptor expression with earlier relapse with distant metastases in oestrogen receptor-positive tumours, especially those of women with more aggressive disease as assessed either by presence of involved lymph nodes or high histological tumour grade. Successful targeting of the type I IGF receptor would abrogate effectively the IGF signal for cell survival. Collectively, our results support the concept that effective combinations of targeted drugs should include abrogation of the activity of the IGF signal transduction pathway.

## Methods

### Cell culture

Breast cancer cell lines: MCF-7, T-47D, ZR-75 and EFM-19 were obtained from the American Type Culture Collection (Manassas, VA) or DMCSZ and cultured routinely in Dulbecco’s modified Eagle’s medium (DMEM) (Sigma, Poole, United Kingdom), supplemented with 10 % foetal calf serum (FCS) and 1 μgml^−1^ insulin in a humidified incubator at 37 °C with 5 % CO_2_.

### Anoikis assay

The non-ionic acid poly(2-hydroxyethyl methacrylate) (poly-HEMA; SIGMA) which inhibits matrix deposition and cell attachment [26] was dissolved in 99 % ethanol at 10 mgml^−1^. Twelve-well tissue culture plates were coated twice with 0.5 ml poly-HEMA solution, allowed to dry, washed with phosphate-buffered saline (PBS) and stored at 4 °C.

Cells were trypsinised, 40 × 10^4^ cells added to 35-mm-diameter poly-HEMA-coated wells and cultured in maintenance medium or in serum-free, phenol red-free DMEM in the absence or presence of IGF-1 for different lengths of time. Cells were recovered, centrifuged, and lysed for protein analysis. Cells were incubated without and with 20 μM PI3-kinase inhibitor, LY294002, (Sigma), 100 nM Akt inhibitor, GSK 690693, (SYN│thesis med chem Pty Ltd., Cambridge, United Kingdom) or 6 μM MEK 1 and MEK2 inhibitor, UO126, (SIgma) for 30 min prior to addition of IGF-1. The type I IGF receptor inhibitory antibody figitumumab [16] was from Pfizer Inc. (Tadworth, United Kingdom). Cells were centrifuged, and lysed for protein analysis.

Activation of caspase 3 was measured with the BD Pharmingen FITC-conjugated active caspase 3 apoptosis kit I (BD Biosciences, Oxford, United Kingdom). Cells were added to 16-mm-diameter poly-HEMA-coated wells at 40 × 10^4^ cells/well in 2 ml of serum-free medium in the absence or presence of 50 ng/ml IGF-1 and incubated for 5 h. Cells were recovered, centrifuged, washed in PBS and, resuspended in 0.2 ml Cytofix/Cytoperm solution and incubated on ice for 20 min. Cells were then washed twice with 0.2 ml Perm/Wash solution, resuspended in 100 μl Perm/Wash solution with 8 μl and FITC-conjugated anti-activated caspase 3 antibody, protected from light and incubated for 30 min. Cells were rewashed, centrifuged and resuspended in 400–500 μl Perm/Wash solution. Fluorescence was measured with a BD FACScan flow cytometer. Excitation was at 488 nm and emission was measured at 530 ± 15 nm.

### Knockdown of the type I IGF receptor

The sequence of the double-stranded short interfering RNAs (siRNA) designed to target the type I IGF receptor mRNA was 5’-CGACUAUCAGCAGCUGAAGTT-3’, and equivalent non-silencing scrambled sequence was 5’-UUCUCCGAACGUGUCACGUdTdT-3’ (Sigma). The siRNA duplex was mixed with RNA iMax (Invitrogen, Paisley, United Kingdom) in serum-free DMEM and incubated 30 min at room temperature. MCF-7 cells were trypsinised, resuspended in maintenance medium at a density of 25 × 10^4^ cellsml^−1^, mixed with the transfection medium and added to a 25 cm^2^ flask. Cells were tested in anoikis assays or lysed for protein analysis, after incubation for 48 h or 96 h, respectively.

### IGF-1 stimulated protein phosphorylation

Cells were added to 16-mm-diameter wells at a density of 15 × 10^4^ cells/well for MCF-7 and ZR-75 cells, and 20 × 10^4^ cells/well for EFM-19 cells. Cells were allowed to attach for 24-48 h and withdrawn from stimulatory factors in serum by culture for 48 h in growth factor-depleted medium, comprising phenol red-free DMEM and 10 % dextran-coated, charcoal-treated serum. Medium was changed daily. Cells were incubated with different concentrations of IGF-1 for 15 min, lysed and analysed by Western transfer [12].

### Western transfer analysis

Cells were lysed in radioimmunoprecipitate (RIPA) buffer which comprised 50 mM Tris–HCl pH 7.5, 150 mM NaCl, 1 mM EDTA, 1 % NP-40 (v/v) and 0.25 % sodium deoxycholate (w/v, 1 μgml^−1^ pepstatin, 1 μgml^−1^ aprotinin, 1 μgml^- 1^ leupeptin, 2 mM sodium orthovanadate, 2 mM sodium fluoride and 2 mM phenyl methyl sulphonyl fluoride. Protein concentrations were measured with a bicinchonic acid assay (Thermo Scientific, Loughborough, United Kingdom). Equal amounts of protein were separated by electrophoresis on denaturing 12 % polyacrylamide gels and transferred to a Westran 0.45 μm nitrocellulose membrane (VWR, Leicestershire, United Kingdom) [50]. Membranes were incubated with specific antibodies against: cleaved poly-(ADP-ribose) polymerase (PARP) (#9541), type I IGF receptor (#3027), phosphorylated type I IGF receptor Tyr1135&1136 and insulin receptor Tyr1150&1151. (#3024), Akt (#9272), phosphorylated Akt Ser473 (#4060), FAK (#3285), phosphorylated FAK Tyr397 (#3283), ERK1 and ERK 2 (#9102), phosphorylated ERK 1 and ERK2 Thr 202 or Tyr 204 (#4370), Bad (#9292), phosphorylated Bad Ser136 (#4366), GSK3β (#9315), phosphorylated GSK3β Ser9 (#9323) (Cell Signaling Technologies, Hitchin, United Kingdom), IRS-1 (sc-7200), phosphorylated IRS-1 Tyr632 (sc-17196-R), and GAPDH (sc-25778) (Santa Cruz Biotechnology, Heidelberg, Germany). Membranes were incubated with horseradish peroxidase conjugated secondary antibodies followed by enhanced chemiluminescence with SuperSignal West Dura Substrate (Thermo Scientific) and exposure to SuperRX X-ray film (Fujifilm, Bedford, United Kingdom). The intensity of the protein bands was quantified by densitometry with Labworks 4.0 software (UVP Inc., Cambridge, United Kingdom).

### Statistics

For the western transfer images, a representative example is shown. Data were normalized and expressed as a percentage of the maximum cleaved PARP or activated signal transduction protein detected. Results are expressed as means ± S.E.M. Differences between groups were tested by analysis of variance, paired or unpaired t-test; p < 0.05 was considered statistically significant. Experiments were replicated at least thrice. The association between type I IGF receptor expression and time to relapse with distant metastasis was analysed by the log rank test on data from The Cancer Genome Atlas.

## Abbreviations

MAP-kinase: mitogen-activated protein kinase
IGF: insulin-like growth factor
siRNA: small interfering RNA
FAK: focal adhesion kinase
poly-HEMA: poly(hydroxyethyl methacrylic)
RIPA: radioimmunoprecipitate
